# P-84. Microbiologic and histologic concordance of pedal osteomyelitis and association with infection progression

**DOI:** 10.1093/ofid/ofae631.291

**Published:** 2025-01-29

**Authors:** Marissa Maier, Kristina L Bajema, Julieta Madrid-Morales, Robert Bosch

**Affiliations:** VA Portland Health Care System/Oregon Health and Sciences University, Portland, Oregon; Veterans Affairs Portland Health Care System, Oregon Health and Science University, Portland, Oregon; Veterans Affairs Portland Health Care System, Oregon Health and Science University, Portland, Oregon; Portland VA, Portland, Oregon

## Abstract

**Background:**

Diabetes-related infections and peripheral artery disease are the leading causes of foot amputation worldwide, resulting in significant morbidity and mortality. Clinical management practices vary widely. We sought to describe the concordance of microbiology and pathology results among persons undergoing forefoot or midfoot bone resection.

**Methods:**

We conducted a retrospective cohort study of adults in Veteran’s Administration (VA) care who underwent forefoot or midfoot bone debridement at VA Portland from January 1, 2017 through December 31, 2019. We used VA’s Corporate Data Warehouse, a centralized database with comprehensive demographic and clinical data, and obtained additional clinical information through chart review. We excluded patients who underwent hindfoot debridement, or intervention on the ipsilateral foot within the preceding year. We defined positive pathology as acute or chronic osteomyelitis and positive microbiology as any bacterial growth from the resection clean margin. Descriptive analyses included calculation of the proportion of individuals with both microbiology and pathology results available versus microbiology or pathology results alone.

**Results:**

From January 1, 2017 – December 31, 2019, there were 379 eligible resections performed among 343 patients, who were 97% male, 82% white, and with median age 69 years. Of the 379 resections, 44% (N=167) had both microbiology and pathology results, 11% (N=44) had microbiology alone, and 45% (N=172) had pathology alone.

Of resections with both microbiology and pathology results, 87% (146/167) had bacterial growth and 58% (97/167) had osteomyelitis on pathology; bacterial growth was present in 90.7% and 82.6% of specimens with and without osteomyelitis on pathology, respectively. Of those with microbiology alone, 93% (41/44) had bacterial growth. Of those with pathology alone, 50% (87/172) had osteomyelitis on pathology.

**Conclusion:**

Similar proportions of bacterial growth were observed whether pathology evidence of osteomyelitis was present. The utility of microbiology results alone to inform antibiotic treatment decisions requires further study.

**Disclosures:**

**All Authors**: No reported disclosures

